# Chemo-Immunotherapy: A New Trend in Cancer Treatment

**DOI:** 10.3390/cancers15112912

**Published:** 2023-05-25

**Authors:** Christian Sordo-Bahamonde, Seila Lorenzo-Herrero, Ana P. Gonzalez-Rodriguez, Alejandra Martínez-Pérez, Juan P. Rodrigo, Juana M. García-Pedrero, Segundo Gonzalez

**Affiliations:** 1 Department of Functional Biology, Immunology, Universidad de Oviedo, 33006 Oviedo, Spain; christiansbl87@gmail.com (C.S.-B.); seilalorenzoherrero@gmail.com (S.L.-H.); uo202446@uniovi.es (A.M.-P.); 2 Instituto Universitario de Oncología del Principado de Asturias (IUOPA), 33006 Oviedo, Spain; anapilargonzalez@gmail.com (A.P.G.-R.);; 3 Instituto de Investigación Sanitaria del Principado de Asturias (ISPA), 33011 Oviedo, Spain; 4 Department of Hematology, Hospital Universitario Central de Asturias (HUCA), 33011 Oviedo, Spain; 5 Department of Otolaryngology-Head and Neck Surgery, Hospital Universitario Central de Asturias (HUCA), 33011 Oviedo, Spain; 6 Centro de Investigación Biomédica en Red de Cáncer (CIBERONC), Instituto de Salud Carlos III, 28029 Madrid, Spain

**Keywords:** immunotherapy, chemotherapy, immune checkpoints, PD-1, T cell, NK cell

## Abstract

**Simple Summary:**

Chemoimmunotherapy is an emerging treatment option for cancer that combines traditional chemotherapy with immunotherapy. This approach aims to increase the efficacy of cancer treatment by simultaneously targeting cancer cells through chemotherapy and boosting the immune system’s ability to fight cancer through immunotherapy. Several studies have shown promising results after using chemoimmunotherapy to treat various types of cancer, including melanoma and lung cancer. However, the optimal dosing, timing, and sequencing of these treatments still require further investigation. In this review, we summarize recent advances and future directions in the field of chemoimmunotherapy in the clinical management of patients with cancer.

**Abstract:**

Chemotherapy has been the basis of advanced cancer treatment for decades. This therapy has largely been considered immunosuppressive, yet accumulated preclinical and clinical evidence shows that certain chemotherapeutic drugs, under defined conditions, may stimulate antitumor immunity and potentiate immune checkpoint inhibitor (ICI)-based therapy. Its effectiveness has been highlighted by recent regulatory approvals of various combinations of chemotherapy with ICIs in several tumors, particularly in some difficult-to-treat cancers. This review discusses the immune modulatory properties of chemotherapy and how they may be harnessed to develop novel chemo-immunotherapy combinations. It also highlights the key determinants of the success of chemo-immunotherapy and provides an overview of the combined chemo-immunotherapies that have been clinically approved.

## 1. Chemotherapy and Immunotherapy: Friends or Foes?

Chemotherapy has been the cornerstone of cancer treatment for over 70 years. In the last decade, ICIs have revolutionized cancer treatment, becoming the frontline therapy for many cancers. In some tumors, such as melanoma, renal cell carcinoma, and others, immunotherapy has largely replaced chemotherapy owing to its clinical benefits and toxic profile, and generally being more manageable and less severe than chemotherapy and radiotherapy [1]. Nevertheless, despite this impressive clinical revolution, the rate of response to immune checkpoint blockade monotherapy is usually around 20% across solid tumors due to primary and acquired resistance to ICIs [2]. The identification of novel biomarkers to discriminate the best responders and the combination of ICIs with other therapeutic modalities are promising avenues to improve their clinical response and patient outcomes.

Cytotoxic chemotherapy has widely been regarded as immunosuppressive, since it causes dose-dependent myelosuppression, thereby suggesting an antagonistic effect with immunotherapy. Nevertheless, accumulated preclinical and clinical evidence has shown that certain chemotherapeutic drugs may act, under defined conditions, as strong adjuvants for enhancing antitumor immunity and, as a result, may potentiate immunotherapy [3]. Accordingly, more than 200 clinical trials combining PD-1/PD-L1 blockade with chemotherapy have already been completed, and several chemo-immunotherapy combinations have recently been clinically approved owing to their improvement in patient survival, with generally expected safety profiles of the known toxicities of each agent [4,5]. In this review, we discuss the mechanisms and conditions in which chemotherapy may stimulate antitumor immunity, and how this may be harnessed to improve the clinical effectiveness of ICIs.

## 2. Immune Checkpoints

Immune checkpoints are crucial regulators of the activation of T cells that play a physiological role in preventing anti-self-responses and autoimmunity. In advanced cancers and chronic viral infections, chronic T cell stimulation induces and up-regulates the expression of inhibitory immune checkpoints, including Programmed Cell Death 1 (PD-1) and Cytotoxic T-Lymphocyte Associated Protein 4 (CTLA-4), displaying an exhausted phenotype characterized by decreased proliferation, differentiation, and survival. T cell exhaustion limits unwanted immune responses in chronic viral infections, but hinders antitumor immunity in advanced cancers. Monoclonal antibodies targeting inhibitory immune checkpoints, including CTLA-4, PD-1, Programmed Cell Death 1 Ligand 1 (PD-L1), and Lymphocyte-Activation Gene 3 (LAG-3) capable of interfering with negative signals provided by these molecules have revolutionized cancer treatment. Despite their impressive clinical results, the rate of response to ICI monotherapies is far from being satisfactory, and a majority of patients with cancer have failed to exhibit clinical benefits from these therapies [2]. Still, decades of chemotherapeutic treatment of cancer have shown that, with rare exceptions, single drugs targeting individual steps of carcinogenesis have demonstrated limited capability to cure due to the heterogeneity and complexity of advanced cancers. Combining different drugs and therapeutic modalities is an obvious strategy to improve patient outcomes [2,6]. 

## 3. The Rationale behind the Combination of Chemotherapy with Immune Checkpoint Inhibitors

Cytotoxic drugs directly kill tumor cells and/or hinder their proliferation via multiple mechanisms including inducing DNA damage, inhibiting DNA replication, and/or preventing mitosis. Chemotherapeutic drugs in monotherapy have shown, with rare exceptions, limited efficacy; however, combination chemotherapy targeting multiple steps in carcinogenesis has been found to be a more effective strategy and, hence, has been widely extended and applied for cancer treatment. Combination regimens may provide a meaningful advantage over monotherapy, by maximizing cancer elimination within the range of tolerated toxicity, targeting a broader range of tumor cells with different genetic and epigenetic abnormalities among a heterogeneous tumor population, and also limiting or slowing the development of drug resistance. 

Conventional chemotherapy has a cytotoxic and cytostatic effect on healthy proliferating cells, especially on hematopoietic cells, causing myelosuppression. This suggests an antagonistic effect between chemotherapy and immunotherapy. In fact, some immunosuppressive drugs used to treat autoimmune diseases or to prevent transplant rejection are chemotherapeutics. Nevertheless, mounting evidence shows that the activation of host immunity decisively contributes to the efficacy of certain cytotoxic drugs; under defined conditions, they may display an immune stimulatory effect, providing an opportunity for their combination with immunotherapy [3,7,8]. The rationale behind this combination lies in the fact that immunotherapy has the capability to eliminate disseminated and metastatic cancer, while it is less effective in eradicating a solid tumor mass [6]. Chemotherapy may potentiate the efficacy of immunotherapy because it has the ability to debulk the primary tumor mass, decreasing the number of cells that should therefore need to be eliminated by immune cells, and also reducing the immunosuppressive factors produced by cancer cells. Additionally, certain chemotherapeutic drugs may directly stimulate antitumor immunity, which may be particularly relevant in “cold” tumors with low effector T cell infiltration within the tumor mass.

## 4. Chemotherapy May Boost Antitumor Immunity

**Figure 1:**
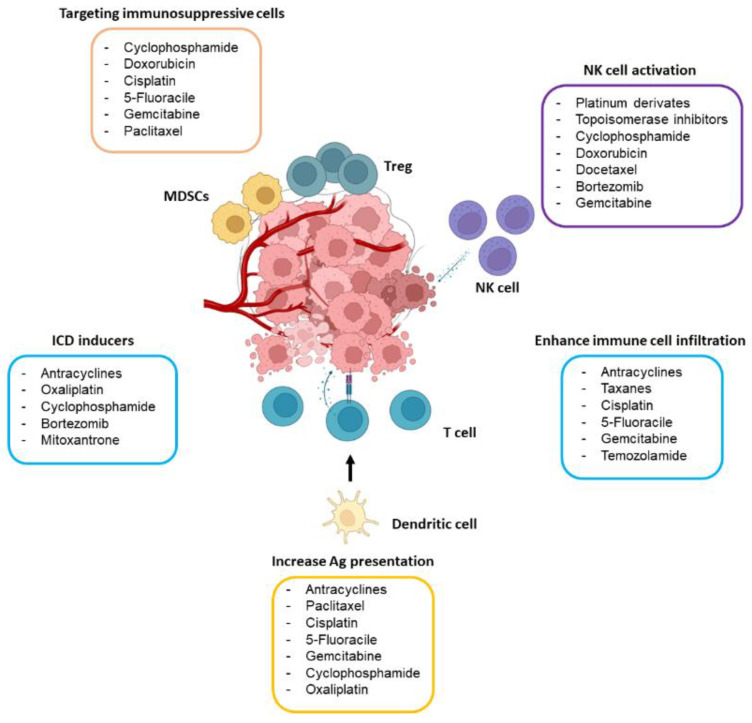
Main immunomodulatory effects of chemotherapeutic drugs. The drugs included in the figure may boost antitumor immunity by targeting immunosuppressive immune cells (mostly Tregs and MDSCs), activating NK cells, causing ICD, and stimulating antigen (Ag) presentation through dendritic cells and T cell activity. The dose of the drug seems to play a crucial role in its capability to stimulate the immune system.

Abundant preclinical evidence demonstrates that the efficacy of certain chemotherapeutic agents is higher in immunocompetent mice than in their immunodeficient counterparts [9]. In good agreement, diverse studies have reported that common chemotherapeutic drugs may induce, in a dose- and schedule-dependent manner, antitumor immunity, mainly through the activation of effector T cells and NK cells and by specifically targeting the immunosuppressive tumor microenvironment (TME). In this section, we discuss the main immunomodulatory mechanisms underlying the action of chemotherapy (Figure 1).

### 4.1. Chemotherapy Activates T Cell Response 

**Figure 2:**
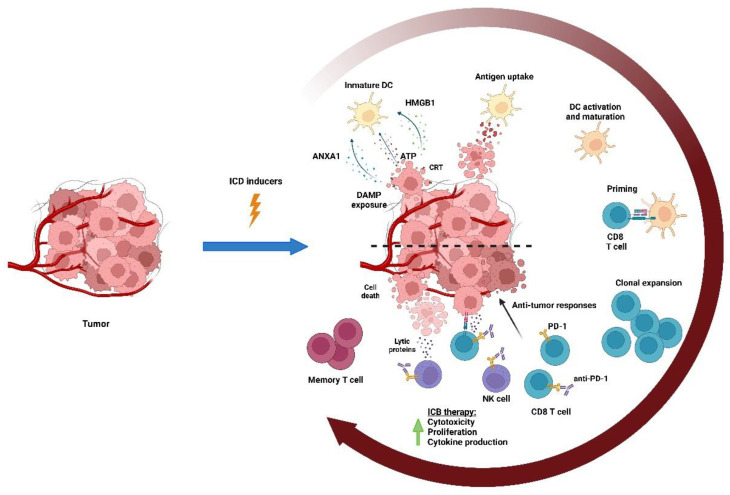
Immunogenic cell death (ICD). Chemotherapeutic drugs may induce the immunogenic death of tumor cells, which results in a CD8 T cell-mediated response against tumor antigens expressed by the dying cells. ICD leads to the exposure and release of DAMPs into TME, which are mainly recognized by DCs. Some DAMPs, including ATP and annexin 1 (ANXA1), induce the recruitment of DCs; others, such as calreticulin, are expressed on the membrane of tumor cells acting as an “eat-me signal” enabling their uptake by DCs. The release of HGMB1, type I interferons, and several cytokines and chemokines culminate in the maturation of DCs and the recruitment and activation of antitumor CD8 T cells that mediate the response against the tumor and generate long-term immune memory.

The type of cell death caused by cytotoxic chemotherapy is a determinant factor for triggering immunity or immune tolerance. Immunogenic cell death (ICD) is a modality of regulated cell death that results in cytotoxic lymphocytes (CTL)-mediated responses against antigens expressed by dying cells, ultimately triggering immunological memory (Figure 2) [3,7,8]. ICD is elicited by several cancer therapies, including radiotherapy and some chemotherapeutic drugs, such as anthracyclines, taxanes, cyclophosphamide, bortezomib, crizotinib, oxaliplatin, and other platinum-derivates (however, cisplatin is not a bona fide ICD-inducer). ICD is a potent endogenous immune adjuvant to the host innate immune system through the exposure and release of danger-associated molecular patterns (DAMPs) into the TME that are recognized by pattern recognition receptors expressed by antigen-presenting cells, mostly dendritic cells (DCs). Some DAMPs, including adenosine triphosphate (ATP) and annexin 1, enable the recruitment and chemotaxis of DCs; others, such as calreticulin, are exposed on the cell membrane acting as an “eat me signal” for the engulfment of the dying cell by DCs [8]. The release of high-mobility group protein B1 (HMGB1) and the secretion of multiple cytokines, including type I interferons, culminates in the maturation of the DCs and the recruitment and activation of the CD8 T cell-mediated immune response against the tumor cells [8]. This cascade of events promotes immune cell infiltration, shifting the tumors from “cold” to “hot” phenotypes [10]. 

Growing preclinical evidence has shown that the immune stimulatory potential of ICD-induced drugs may be harnessed to improve the efficacy of the PD-1/PD-L1 blockade [7,11,12,13,14,15]. In patients with HER2-negative unresectable gastric and gastro-esophageal junction adenocarcinomas, the combination of oxaliplatin-based chemotherapy with nivolumab significantly improved patients’ survival [16]. Adding trastuzumab and chemotherapy (5-fluorouracil and cisplatin or capecitabine and oxaliplatin) to the PD-1 blockade resulted in a significant improvement in the objective response rate (ORR) (from 51.9% to 74.4%) in unresectable or metastatic HER2+ gastric or gastro-esophageal junction adenocarcinoma [17]. The majority of metastatic triple-negative breast cancer (TNBC) patients showed no benefit from a PD-1/PD-L1 blockade in a phase II clinical trial (TONIC). However, the combination of doxorubicin with nivolumab resulted in 35% ORR, which was superior to cisplatin plus nivolumab (23%) [18]. Interestingly, doxorubicin and cisplatin treatment induced the upregulation of genes involved in the T cell cytotoxicity pathway, thereby providing a link between the clinical activity of these agents and their capacity to regulate systemic immunity. Nevertheless, a direct consequence of the induction of ICD is the upregulation of PD-L1 expression in many cancers and in myeloid cells, thus exerting a negative immunomodulatory effect, and altogether providing another justification for their combination with a PD-1/PD-L1 blockade [7,8,18,19]. 

Non-lethal stress elicited by certain cytotoxic drugs may also activate T cells and render tumors more susceptible to T cell killing. For instance, treatment with paclitaxel induced the infiltration of CTLs in a mouse model of ovarian cancer [19] and patients with breast cancer [20], as well as 5-fluorouracil in a murine model of breast cancer [21] and temozolomide in models of melanoma [22]. Multiple cytotoxic drugs have been shown to enhance DC maturation and activation, antigen presentation, and T cell activation, mainly in vitro [23,24,25]. Cisplatin and doxorubicin have been shown to sensitize the most resistant colon cancer cell lines to TRAIL-induced cell death [26], and cisplatin, doxorubicin, and paclitaxel sensitize tumor cells to CTLs by making tumor cells permeable to granzyme B in mice [27]. 

### 4.2. Chemotherapy Dampens the Immunosuppressive Tumor Microenvironment 

Advanced cancers progressively accumulate immunosuppressive cells in their TME, mostly regulatory T (Treg) cells and myeloid-derived suppressor cells (MDSCs) that remain a major barrier hindering effective antitumor immunity. Low doses of several cytotoxic drugs can selectively deplete both circulating and tumor-infiltrating Tregs concomitantly, stimulating antitumor immunity (Figure 2). Interestingly, this effect is particularly well documented with low-dose cyclophosphamide [28]. Mechanistically, Tregs lack the expression of cyclophosphamide-extruding transporter ABCB1, being more sensitive to cyclophosphamide than effector immune cells [29]. It is worth mentioning that cyclophosphamide at a higher dose also induces ICD. Multiple chemotherapeutic agents, including cyclophosphamide, cisplatin, paclitaxel, 5-fluorouracil, gemcitabine, and doxorubicin, selectively eliminate MDSCs in multiple mouse tumor models, resulting in immune recovery and tumor regression [30,31,32,33,34]. However, recent reports suggest that, under certain conditions, chemotherapy might also induce the accumulation of MDSCs in TME [35,36,37]. For example, certain cytotoxic drugs such as cyclophosphamide and melphalan may cause an increase in MDSC infiltration due to the inflammatory response triggered by chemotherapy [36]. Thus, the effects of chemotherapy on MDSCs can vary depending on several factors, including the chemotherapeutic agent, dosage, and timing. Nevertheless, the clinical relevance of this preclinical evidence in cancer patients remains to be established [38].

### 4.3. Chemotherapy Activates NK Cells

NK cells are cytotoxic innate immune cells that play a relevant role in cancer immunosurveillance and immunotherapy, particularly in hematological cancers and metastasis [39]. NK cells can eliminate malignant tumors in a non-MHC and non-tumor antigen-restricted manner through an array of activating (i.e., NKG2D, DNAM-1, NCRs) and inhibitory receptors (i.e., KIRs, NKG2A-CD94) that detect changes in the expression of their ligands during viral infection and malignant transformation. Mounting preclinical evidence shows that the DNA damage response pathway initiated by ATM, ATR, and p53, induced by multiple genotoxic drugs, triggers tumor cells to express ligands for the NKG2D receptor. This upregulation promotes NK cell-mediated cytotoxicity and IFN-γ release, which subsequently favors the upregulation of MHC class I molecules on tumor cells, sensitizing them to CTLs (Figure 1) [40]. Similarly, hyperdiploid-inducing chemotherapeutic agents, including cytochalasin D, nocodazole, and docetaxel, strongly upregulate the tumor expression of NKG2D and DNAM-1 ligands, rendering tumor cells more susceptible to NK cell-mediated lysis [41]. In patients with lung cancer, low-dose gemcitabine enhanced NK cell-mediated cytotoxicity [42], and a maintained administration of low-dose cyclophosphamide, referred to as metronomic dose (see Glossary), enhanced NK cell activity in end-stage cancer patients [43].

PD-1 is not expressed in peripheral blood NK cells from most healthy individuals; however, in the context of cancer, its expression is induced in peripheral and tumor-derived NK cells, dampening antitumor immunity, which has been correlated with poor prognosis in multiple cancer patients [44,45]. Interestingly, the response to PD-1 blockade may be enhanced by the increased number and activation of NK cells, thereby improving the clinical effectiveness, particularly in MHC class I-defective tumors [46,47,48,49]. It is worth mentioning that some tumor cells can induce PD-L1 expression on NK cells via AKT signaling, and the PD-L1 blockade results in enhanced NK cell activity and tumor regression [49]. This provides a potential explanation as to why some patients lacking PD-L1 expression in cancer cells still respond to anti-PD-L1 therapy. Collectively, accumulating evidence suggests a relevant contribution of NK cells to the clinical success of ICIs and, in this scenario, chemotherapy may improve their effectiveness through the activation of this immune subset.

## 5. Determinants of the Success of Chemo-Immunotherapy

### 5.1. The Right Dose of Chemotherapy

Chemotherapy drugs cause dose-dependent myelosuppression and, in the clinic, are usually administered at the maximum tolerated dose causing immunosuppression. Despite variable clinical results, metronomic chemotherapy is a promising alternative to the conventional dosage that may have a beneficial effect on TME by inhibiting tumor angiogenesis and boosting antitumor immunity, while avoiding toxicity caused by maximum-tolerated dose treatments [50]. The underlying mechanism is far from being elucidated, but maximum tolerated dose regimens are associated with a depletion of effector immune cells, including CD4 and CD8 T cells, NK cells, and γδT cells, whereas low-dose regimens selectively target immunosuppressive Tregs and MDSCs, ameliorate T cell exhaustion, promote the maturation and activation of DCs, and concomitantly activate the NK and T cell-mediated antitumor immunity [33,51,52,53,54,55,56]. Standard regimens, but not metronomic doses of temozolomide or paclitaxel, have abrogated the survival advantage provided by a PD-1 blockade in murine glioma and TNBC models, respectively [53]. Metronomic gemcitabine in models of non-small-cell lung carcinoma (NSCLC) and low-dose cyclophosphamide in neuroblastoma have led to the increased efficacy and diminished toxicity of the PD-1 blockade due to reduced tumor angiogenesis dampening Tregs and enhancing the T cell effector response [57]. Along these lines, metronomic oxaliplatin and pemetrexed together with a PD-1 blockade have successfully activated T cell immunity, eliciting tumor-specific long-term immune memory in colon cancer models [58]. Similar results have been reported for combined metronomic chemotherapy with a multi-peptide vaccine and anti-PD-1 checkpoint inhibition in melanoma in vivo [59]. These preclinical data suggest that the balance between active antitumor immunity and tumor elimination with less toxicity could be critical for the success of chemo-immunotherapy. In clinical settings, chemotherapy is conventionally administered at a maximum tolerated dose, and the effect of metronomic chemotherapy has not yet been well-established [28,60]. This is particularly true for older patients, who are under-represented in current standardized clinical trials, and in whom a metronomic dose may ameliorate its adverse effects.

### 5.2. The Timing of Chemo-Immunotherapy

TME is a key determinant of ICI responsiveness, and dynamically changes alongside tumor progression. A pronounced synergistic effect between immunotherapy and chemotherapy may be achieved in mouse models wherein the immune system of the mice is intact. Nevertheless, current ICIs are usually administered to patients with advanced cancer, who exhibit a deteriorated immune system due to immunoediting and chemotherapy treatment. Theoretically, immunotherapy administered to patients in earlier stages of the disease, with less deteriorated immunity and before a myeloablative chemotherapy treatment, would be more likely to cause a durable immunity than that caused by most current regimens [61]. Likewise, first-line durvalumab in combination with etoposide plus platinum in treatment-naïve early-stage small-cell lung cancer (SCLC) showed an improvement in overall survival (OS) compared with chemotherapy alone [62]. A recent meta-analysis based on 12 phase-III clinical trials with 9236 metastatic NSCLC patients reported that the addition of chemotherapy to ICIs enhanced their treatment efficacy as a first-line treatment [63]. Nevertheless, this approach could have the disadvantage of exposing patients who would have responded to monotherapy to unnecessary toxicity.

### 5.3. The Sequence of Chemo-Immunotherapy

Chemotherapy and immunotherapy are administered concurrently in the vast majority of clinical trials. Still, the sequence of their administration may meaningfully affect outcomes [64]. For instance, ipilimumab (anti-CTLA-4 antibody) administered after carboplatin and paclitaxel (but not concurrent administration) is associated with improved immune-related progression-free survival (PFS) in SCLC compared with chemotherapy alone [65]. By contrast, patients with metastatic melanoma who progress after PD-1 therapy benefit from the subsequent addition of chemotherapy [66]. Therefore, a rational timing selection is susceptible to becoming a cornerstone of chemo-immunotherapy success; intuitively, immunotherapy is more likely to work when administered before myeloablative chemotherapy regimens. Contrarily, non-myeloablative chemotherapy using drugs with immune stimulatory properties (i.e., causing ICD or a metronomic dose) are more likely to work before immunotherapy. Of note, doxorubicin and oxaliplatin, which are particularly efficient in promoting immune responses, are promising partners for administration before chemotherapy [67]. Enhancing lymphocyte recovery using immunomodulatory drugs or cytokines or minimizing chemotherapy-induced damage to the immune system may potentiate ICIs, and may be an alternative to a maximum tolerated dose of chemotherapy currently used in clinical practice. Unfortunately, few clinical trials have tried to systematically identify the optimal conditions for chemo-immunotherapy, and no consensus has yet been achieved regarding the right dose, timing, and sequence of chemo-immunotherapy combinations that may maximize their clinical benefits.

## 6. Overview of Clinically Approved Chemo-Immunotherapy Combinations

Combining ICIs with standard-of-care chemotherapy has been successful in the treatment of several tumors, particularly in some difficult-to-treat cancers with limited risks of overlapping toxicities between individual drugs (Table 1). The first and foremost success of chemo-immunotherapy has been achieved in lung cancer. Unprecedented efficacy was observed with the addition of standard chemotherapy to a PD-1 blockade with pembrolizumab, reducing the risk of death by half compared to chemotherapy alone in non-squamous NSCLC (OS at 12 months 69.2% vs. 49.4%) [4], and in squamous NSCLC regardless of tumor PD-L1 expression status (OS 15.9 vs. 11.3 months) [68]. A combination of chemotherapy with the anti-PD-L1 antibody atezolizumab or anti-PD-1 antibody nivolumab plus ipilimumab also improved patient survival in NSCLC [69,70]. Notably, neoadjuvant nivolumab combined with chemotherapy resulted in a significant improvement in event-free survival (EFS) and a pathological complete response (CR) in patients with resectable NSCLC (24% vs. 2.2%) [71], suggesting that immunotherapy before surgery may enhance antitumor T cell immunity, favoring the rejection of micro-metastases after surgical resection [72]. In SCLC, the addition of standard platinum to the PD-L1 blockade with atezolizumab or durvalumab yielded better results than chemotherapy alone (atezolizumab OS 12.3 vs. 10.3 months; durvalumab OS 13 vs. 10.3 months) [62,73].

TNBC is the breast cancer subtype with the poorest prognosis. However, it is more frequently infiltrated by tumor-infiltrating lymphocytes and more frequently expresses PD-L1 than other subtypes, thus suggesting that ICIs may be a promising therapy for TNBC. In patients expressing PD-L1 with a combined positive score (CPS) ≥ 10, pembrolizumab in combination with chemotherapy significantly reduced the risk of progression or death by 35% (PFS 9.7 vs. 5.6 months), leading to FDA approval [74]. In early-stage TNBC, pembrolizumab added to neoadjuvant chemotherapy displayed a superior therapeutic efficacy compared to chemotherapy alone [75]. Similarly, atezolizumab in combination with nab-paclitaxel showed superior clinical effectiveness and was approved for PD-L1+ metastatic TNBC [76].

The conventional first-line treatment in head and neck squamous cell carcinomas (HNSCC) involves the combination of chemotherapy and the anti-EGFR antibody cetuximab. However, the combination of pembrolizumab with chemotherapy displayed superior efficacy to cetuximab plus chemotherapy, and it has been approved for the first-line treatment of patients with metastatic or recurrent HNSCC [77]. Multiple clinical trials have demonstrated the efficacy of chemo-immunotherapy in digestive tumors (Table 1). For instance, pembrolizumab plus 5-fluorouracil and cisplatin reduced the risk of disease progression or death by 35% in patients with locally advanced or metastatic esophageal or gastroesophageal junction carcinoma [78]. Nivolumab plus chemotherapy showed superior efficacy compared to chemotherapy alone, and was approved for first-time treatment for advanced gastric, gastroesophageal junction, or esophageal adenocarcinomas [16], and patients with unresectable advanced or metastatic esophageal squamous cell carcinoma, regardless of PD-L1 status [79]. An OS benefit was observed in patients with metastatic urothelial carcinoma who had completed platinum-based chemotherapy without disease progression and were subsequently maintained with the PD-L1 antibody avelumab (21.1 vs. 14.3 months) [80]. Chemo-immunotherapy combinations were also approved in advanced biliary tract cancer [81], and in cervical cancer [82] (Table 1). Nevertheless, chemo-immunotherapy has not been a panacea for all tumors. Unfortunately, despite this aforementioned remarkable success, clinical studies have not been followed by a deep mechanistic analysis or the identification of predictive biomarkers. This means that it is likely that the drug combinations, the dose, the sequence, and the timing were not optimal in most clinical trials, and it is likely that a certain degree of immune cell toxicity and a lack of synergism hindered the efficacy of the combination of chemotherapy and ICIs.

**Table 1:** FDA-approved chemotherapy and immunotherapy combinations.

Cancer	Line of Therapy	PD-L1 Positivity Criteria	Chemotherapy	ICI	Clinical Benefit	Trial Name
NSCLC-non-squamous	Metastatic, first-line	Regardless of PD-L1 tumor expression	Pemetrexed + carboplatin	Pembrolizumab	OS at 12 m: 69.2% vs. 49.4%. HR 0.49; [95% CI 0.38–0.64]; *p* < 0.00001	Keynote-189 [4]
NSCLC-squamous	Metastatic, first-line	Regardless of PD-L1 tumor expression	Carboplatin + paclitaxel/ nab paclitaxel	Pembrolizumab	OS: 15.9 vs. 11.3 m. HR 0.64; [95% CI 0.49–0.85]; *p* = 0.001	Keynote-407 [68]
NSCLC-non-squamous	Metastatic, first-line	Regardless of PD-L1 tumor expression	Carboplatin + paclitaxel + bevacizumab	Atezolizumab	OS: 19.2 vs. 14.7 m. HR 0.78; [95% CI 0.64–0.96]; *p* = 0.01	IMpower 150 [69]
NSCLC-non-squamous	Metastatic, first-line	Regardless of PD-L1 tumor expression	Carboplatin + nab paclitaxel	Atezolizumab	OS: 18.6 vs. 13.9 m. HR 0.8; [95% CI 0.64–0.99]; *p* = 0.03	IMpower 130 [5]
NSCLC	Metastatic, first-line	Regardless of PD-L1 tumor expression	Platinum doublet	Nivolumab + ipilimumab	OS 15.6 vs. 10.9 m; HR 0.69; [95% CI 0.55–0.80]; *p* = 0.00065	CheckMate-9LA [70]
NSCLC	Neoadjuvant	Regardless of PD-L1 tumor expression	Platinum-based chemotherapy	Nivolumab	EFS 31.6 vs. 20.8 m. HR 0.63; [97.3% CI, 0.43–0.91]; *p* = 0.005. pCR 24.0% vs. 2.2%. OR: 13.9; [99% CI, 3.4–55.7]; *p* < 0.001	Checkmate-816 [71]
NSCLC	Metastatic	PD-L1 expression on ≥1% of tumor cells	Platinum-based chemotherapy + tremelimumab	Durvalumab	Reduced the risk of death by 23% HR 0.77; [95% CI 0.65 to 0.92]; *p* = 0.00304	POSEIDON Phase III trial [83]
NSCLC	Metastatic, first-line	Regardless of PD-L1 tumor expression	Adjuvant treatment following surgical resection and platinum-based chemotherapy	Pembrolizumab	Reduced the risk of disease recurrence or death by 27%; HR 0.73; [95% CI, 0.60 to 0.89]	KEYNOTE-091 [84]
SCLC	Extensive stage, first-line	Regardless of PD-L1 tumor expression	Carboplatin + etoposide	Atezolizumab concurrent and maintenance	OS: 12.3 vs. 10.3 m. HR 0.70; [95% CI 0.54–0.91]; *p* = 0.006	IMpower 133 [73]
SCLC	Extensive stage, first-line	Regardless of PD-L1 tumor expression	Carboplatin + etoposide	Durvalumab	OS: 13 vs. 10.3 m. HR 0.73; [95% CI 0.59–0.91]; *p* = 0.0047	CASPIAN [62]
HNSCC	Metastatic first-line	Regardless of PD-L1 tumor expression	Platinum + 5-FU or platinum + 5-FU + cetuximab	Pembrolizumab	OS: 13.6 vs. 10.4 m. (CPS ≥ 1) HR 0.65; [95% CI 0.53–0.80]; *p* < 0.03	Keynote-048 [77]
Esophagus cancer	Metastatic, first-line	Regardless of PD-L1 tumor expression	5-fluorouracil + cisplatin	Pembrolizumab	OS: 12.4 vs. 9.8 m. HR 0.73; [CI 0.62–0.86]; *p* < 0.0001	Keynote-590 [78]
Esophagus cancer	Metastatic, first-line	Regardless of PD-L1 tumor expression	Fluropyrimidine + platinum-based	Nivolumab	OS: 13.2 vs. 10.7 m. HR 0.74; [99.1% CI, 0.58–0.96]; *p* = 0.002	Checkmate 648 [79]
Gastric/ esophagus cancer	Metastatic, first-line	Regardless of PD-L1 tumor expression	Capecitabine + oxaliplatin or leucovorin + fluorouracil + oxaliplatin	Nivolumab	OS: 13.1 vs. 11.1 m. HR 0.71; [98.4% CI 0.59–0.86]; *p* < 0.0001	Check-Mate-649 [16]
Gastric cancer	Metastatic, first-line	Regardless of PD-L1 tumor expression	Trastuzumab + 5-fluorouracil + cisplatin or capecitabine + oxaliplatin	Pembrolizumab	22.7% improvement in OR [95% CI 11.2–33.7]; *p* = 0.00006. CR 11.3% vs. 3.1%	Keynote-811 [17]
TNBC	Metastatic, first-line	PD-L1 + tumor cells (CPS ≥ 10)	Nab paclitaxel or paclitaxel or carboplatin + Gemcitabine	Pembrolizumab	PFS (CPS > 10): 9.7 vs. 5.6 m. HR 0.65; [95% CI 0.49–0.86]; *p* = 0.0012	Keynote 355 [74]
TNBC	Neoadjuvant	Regardless of PD-L1 tumor expression	Carboplatin + paclitaxel, followed by doxorubicin or epirubicin + cyclophosphamide	Pembrolizumab	37% reduction in the risk of disease progression. HR = 0.63; [95% CI, 0.48–0.82]; *p* = 0.0003	Keynote-522 [75]
TNBC	Metastatic, first-line	PD-L1 + tumor cells (≥1%).	Nab paclitaxel	Atezolizumab	OS: 25.0 vs. 15.5 m. PD-L1(+) HR 0.62; [95% CI 0.45–0.86]	IMpassion 130 [76]
Cervical cancer	Metastatic, first-line	Regardless of PD-L1 tumor expression	Paclitaxel + cisplatin or paclitaxel + carboplatin +/− bevacizumab	Pembrolizumab	ORR 68% vs. 50%. Median of duration response 18.0 vs. 10.4 m	Keynote-826 [82]
Biliary tract cancer	Metastatic, first-line	Regardless of PD-L1 tumor expression	Gemcitabine + cisplatin	Durvalumab	Reduced the risk of death by 20% HR 0.80; [95% CI 0.66–0.97]; *p* = 0.021	TOPAZ-1 [81]
Bladder cancer	Metastatic, first-line maintenance	Regardless of PD-L1 tumor expression	Gemcitabine + cisplatin/carboplatin	Avelumab	OS 21.4 vs. 14.3 m; HR 0.69; [95% CI 0.56 to 0.86]; *p* = 0.001	JAVELIN Bladder 100 [80]

NSCLC: Non-Small Cell Lung Cancer; SCLC: Small Cell Lung Cancer; HNSCC: Head and Neck Squamous Cell Carcinoma; TNBC: Triple Negative Breast Cancer; ICI: Immune Checkpoint Inhibitor; OS: Overall Survival; m: month; HR: Hazard Ratio; CI: Confidence Interval; CPS: Combined Positive Score; EFS Event Free survival; pCR: pathological Complete Response.

## 7. Concluding Remarks

Several chemo-immunotherapy combinations have been successful in the treatment of multiple cancers, showing that chemotherapy can stimulate antitumor immunity and potentiate the clinical activity of ICIs. Nevertheless, hundreds of clinical trials have been completed, but only a few of them have succeeded. The development of fruitful chemo-immunotherapy combinations is constrained by our limited understanding of the immunomodulatory properties of chemotherapeutic drugs, and the optimal dose, timing, and sequence of chemo-immunotherapy combinations needed to tip the balance from immunosuppression to immune stimulation. To move forward, these issues should be re-examined in preclinical models and specific clinical trials, where cutting-edge technologies such as spatial transcriptomics and single-cell sequencing hold tremendous potential for advancing our understanding of the TME and its role in immunotherapy. Spatial transcriptomics allows the mapping of gene expression patterns within intact tissue samples, enabling the identification of different cell types and their interactions within the TME. Single-cell sequencing provides insights into the heterogeneity of individual cells, facilitating the characterization of immune cell subsets and their functional states. Integrating these technologies might provide novel insights into the complex interplay between tumor cells, immune cells, and stromal components in the TME in response to chemotherapy, leading to the identification of novel therapeutic targets and biomarkers for ICIs-based therapies. This deeper understanding has the potential to enhance treatment strategies, personalize therapies, and improve patient outcomes in the future. A major challenge in immunotherapy is in improving the preclinical models that may allow the rapid implementation of chemo-immunotherapy advances in clinical settings. The identification of novel biomarkers to predict the candidates who will obtain the greatest benefit from chemo-immunotherapy combinations is also essential. Despite these limitations, the increasing number of ICIs and the vast options for chemo-immunotherapy combinations for different types of cancers suggest an explosion of novel strategies for cancer therapy in the next few years.

## Glossary

Progression-free survival (PFS)	The time from treatment initiation until disease progression or worsening. It may be used as a direct or surrogate measure of clinical benefit for drug approvals.
Pathological complete response (pCR)	Defined as no residual disease after treatment determined by the pathologist.
Partial response (PR)	The decrease in the size of a tumor, or the extent of cancer in the body, in response to treatment.
Overall survival (OS)	The time from treatment to death, with no restriction on the cause of death. It is universally accepted as a direct measure of clinical benefit; however, in some disease areas, surrogate end-points are used to try to reduce the time taken to analyze new treatments.
Overall response rate (ORR)	The proportion of patients who have a partial or complete response to therapy.
Neoadjuvant therapies	Treatments administered before the main therapy, to help reduce the size of a tumor or kill cancer cells that have spread.
Metronomic chemotherapy	A treatment in which low doses of anti-cancer drugs are given on a continuous or frequent, regular schedule (such as daily or weekly), usually over a long time. Metronomic chemotherapy causes less severe side effects than standard chemotherapy.
Event-free survival (EFS)	The time after treatment for cancer when a patient remains free of certain complications or events that the treatment was intended to prevent. It is a term that denotes the possibility of having a particular group of defined events (could be a fracture, some lab test abnormality, a particular kind of progression such as brain metastasis, etc.) after a treatment that is designed to delay or prevent that group of events.
Combined positive score (CPS)	Corresponds to the total number of tumor cells and immune cells (including lymphocytes and macrophages) stained with PD-L1 divided by the number of all viable tumor cells, then multiplied by 100.
Complete response (CR)	The disappearance of all signs of cancer in response to treatment.
Adjuvant therapies	Treatments administered after the primary therapy to try to kill the remaining cancer cells.
